# Vasoactive Intestinal Polypeptide in the Carotid Body—A History of Forty Years of Research. A Mini Review

**DOI:** 10.3390/ijms21134692

**Published:** 2020-06-30

**Authors:** Slawomir Gonkowski

**Affiliations:** Department of Clinical Physiology, Faculty of Veterinary Medicine, University of Warmia and Mazury in Olsztyn, Oczapowski Str. 13, 10-718 Olsztyn, Poland; slawomir.gonkowski@uwm.edu.pl

**Keywords:** vasoactive intestinal polypeptide, carotid body, nerve fibers, innervation

## Abstract

Vasoactive intestinal polypeptide (VIP) consists of 28 amino acid residues and is widespread in many internal organs and systems. Its presence has also been found in the nervous structures supplying the carotid body not only in mammals but also in birds and amphibians. The number and distribution of VIP in the carotid body clearly depends on the animal species studied; however, among all the species, this neuropeptide is present in nerve fibers around blood vessels and between glomus cell clusters. It is also known that the number of nerves containing VIP located in the carotid body may change under various pathological and physiological factors. The knowledge concerning the functioning of VIP in the carotid body is relatively limited. It is known that VIP may impact the glomus type I cells, causing changes in their spontaneous discharge, but the main impact of VIP on the carotid body is probably connected with the vasodilatory effects of this peptide and its influence on blood flow and oxygen delivery. This review is a concise summary of forty years of research concerning the distribution of VIP in the carotid body.

## 1. Introduction

It is commonly known that the carotid body placed in the carotid bifurcation region is an organ consisting of chemoreceptor cells, which are mainly sensitive to changes in O_2_ and CO_2_ partial pressures in blood. They also have the ability to detect hypoxia and can react to acidic pH in the blood [1,2,3,4]. The carotid body may also play other multidirectional functions, including the regulation of metabolism, the maintenance of glucose homeostasis and thermoregulatory processes, among others [5,6,7].

The history of studies on the carotid body reaches back to the year 1743, when this organ was described for the first time [8]. However, the most accurate investigations of the carotid body were performed only in the 20th century. In the 1920s, De Castro described the histological structure of the carotid body. He discovered chemoreceptors and described nerve fibers in this organ [8,9], and in 1959, the carotid body cell ultrastructure was defined using electron microscopy [10]. The next studies showing that the carotid boy is innervated by both afferent and efferent nerve fibers was performed in the 1960s [11,12].

Now, it is known that the carotid body consists of two types of glomus cells: glomus type I and glomus type II cells. The glomus type I cells are peripheral chemoreceptors. They react to hypoxia (a decrease in O_2_ partial pressure) and acid hypercapnia (an increase in CO_2_/H^+^ partial pressure), undergo depolarization and secrete active substances stimulating afferent nerve fibers, which in turn leads to the activation of cardiorespiratory centers in the central nervous system [13]. In turn, the glomus type II cells (sustentacular cells) are similar to glial cells and play supportive functions in chemoreception within the carotid body, mainly through paracrine cell–cell interactions [13,14].

The carotid body is innervated with a dense network of nerve fibers originating in the carotid sinus nerve (a branch of the glossopharyngeal), the superior cervical ganglion of the sympathetic trunk and branches of the vagal nerve [15,16,17]. Studies based on retrograde neuronal tracing have shown that, in rats, 94.5% of afferent neurons supplying the carotid body are localized in the petrosal ganglion, 5.2% is localized in the jugular ganglion and 0.3% is localized in the nodose ganglion [18]. Nerve fibers located in the carotid body are characterized by a relatively high degree of neurochemical differentiation. It is known that they may contain various neuronal active substances and enzymes including tyrosine hydroxylase (an enzyme for the synthesis of catecholamines), nitric oxide, substance P, enkephalins, bombesin, calcitonin gene related peptide, galanin and neuropeptide Y, among others [15,19,20,21,22,23].

Among these substances, vasoactive intestinal polypeptide (VIP) seems to play an important role in the regulation of the carotid body activity (especially in the regulation of the intraorganic blood flow) under physiological conditions and during pathological states. In the late 1970s and early 1980s, the presence of VIP in the carotid body was noted for the first time [24,25,26]. Despite a few reviews concerning the innervation of the carotid body [19,20,27], the work from this time period is entirely dedicated to stating that VIP-positive structures in this organ do not exist. Therefore, the present review is a concise summary of the history of studies and the current knowledge concerning VIP in the carotid body.

## 2. VIP—Structure, Receptors and Activity

VIP belongs to a glucagon/secretin superfamily and is a peptide consisting of 28 amino acids. Besides VIP, this peptide superfamily includes, among others, pituitary adenylate-cyclase-activating polypeptide (PACAP), secretin, growth hormone-releasing factor, histidine isoleucine peptide, glucagon, helodermin and gastric inhibitory polypeptide [28,29,30,31]. VIP was isolated for the first time as a potent vasodilator and hypotensive peptide from the porcine duodenum in 1970 [32]. In spite of the fact that the largest amount of VIP in the living organism has been found in the gastrointestinal tract [33], the presence of this peptide has also been observed in other various internal organs and systems [30,34,35,36]. Firstly, VIP was identified as a neurotransmitter and/or neuromodulator in the central and peripheral nervous systems, both in autonomic and sensory structures, in various types of neuronal cells and in nerve fibers [33,37,38,39,40]. Moreover, the presence of VIP has been described in cells of the immunological system including eosinophils, mast cells and lymphocytes, where this peptide may play the role of a “cytokine-like peptide” [30,40].

At this time, two types of receptors that may be affected by VIP have been described. These receptors are VPAC1 and VPAC2, and they belong to a class of II G protein-coupled receptors. They stimulate cellular adenylyl cyclase activity and the protein kinase A pathway [30,41]. Moreover, it is known that VPAC1 and VPAC2 (depending on the localization) may often participate in the regulation of other cellular pathways, such as pathways involving nitric oxide, phospholipase C, mitogen-activated protein (MAP) kinases, nuclear factor kappa-light-chain-enhancer of activated B cells (NFκβ) and others [30]. Both types of receptors to VIP are widespread in living organisms. Their presence has been described in the gastrointestinal tract, heart, blood vessels and the immunological system [30,42,43]. Moreover, the presence of VIP receptors has also been noted in cells affected by malignant processes, and their number was clearly higher than in the same type of cells under physiological conditions, which strongly suggests the participation of VIP and its receptors in malignant transformation [41].

Due to the fact that VPAC1 and VPAC2 may participate in various cellular pathways [30,41], VIP plays multidirectional roles in the organism. Apart from regulatory functions concerning the proper functioning of neuronal cells [33,42], it is known that this peptide is involved in regulatory processes in the gastrointestinal tract, where it affects the intestinal motility, blood flow, secretory activity and permeability of the intestinal barrier [30,44]. VIP also regulates the activity of many other systems, including the respiratory, excretory, reproductive and cardiovascular systems [30,36,45,46,47]. Important functions of VIP are also the immunomodulatory activity, the influence of endocrine glands and metabolism, the control of insulin release as well as relaxant effects on the muscles and vasodilatory properties [30,31,33,40,48,49].

In light of the previous studies, it is also known that VIP not only regulates the functions of numerous internal organs under physiological conditions but also takes part in various pathological processes. It has been described as an important factor participating in neuroprotective and adaptive reactions in the central and peripheral nervous systems, where it is the factor enhancing the survivability of neuronal cells [33,50]. Moreover, VIP is involved in cell proliferation during cancer [51] as well as shows strong ant-inflammatory properties [52,53]. Some studies suggest the participation of VIP in pathological processes connected with intoxication from mycotoxins and other environmental pollutants [54,55,56].

The multidirectional activity of VIP and especially its anti-inflammatory and neuroprotective properties have caused this peptide to be considered a promising therapeutic factor with two major therapeutic uses: during inflammatory processes and during neurodegenerative diseases [30,49,52,57].

## 3. VIP in Mammalian Carotid Body

Until now, the presence of VIP has been noted in the carotid body of humans and other numerous mammalian species, including rat, mouse, guinea pig, cat, chipmunk and monkey [19,20,27,58]. In the majority of species, VIP has been described in the varicose nerves located mainly in the connective tissue between glomus cell clusters and near blood vessels. The following is a description of these mammalian species, in which the knowledge concerning the distribution of VIP in the carotid body is relatively broader.

### 3.1. Human

In the case of VIP distribution in the human carotid body, the results obtained by different investigators differ from one another and are not entirely clear. The investigations using the radioimmunoassay method have shown that the levels of VIP in the human carotid body (studied in 13 organs removed at routine necropsies) ranged from 4 to 16 pm/g and that an average value amounted to 9 pm/g [59]. The levels of VIP were significantly lower than the concentration of other peptides, including met-enkephalin (an average level of 612 pm/g), leu-enkephalin (162 pm/g), bombesin (73 pm/g), neurotensin (67 pm/g) and substance P (16 pm/g) [59,60]. In turn, the investigations concerning the distribution of VIP in the human carotid body using the immunostaining method have shown (contrary to other mammalian species) the presence of this neuropeptide in type I cells of the carotid body but not in the nerve fibers and neuronal cells [61]. Carotid body parenchymal cells can be divided into three types: light, dark and pyknotic, whereby the first cell type is considered to be active and the other two cell types are considered to be inactive [62]. Smith et al. [61] investigated 23 carotid bodies collected during routine necropsies. They found VIP in both light and dark cells, but in the majority of cases, the labelling against VIP was faint [61]. Clear strong labelling was observed in only eight cases. Similar observations were made by Kubo et al. [62], who also described faint labelling against VIP in the light and dark cells of the human carotid body, whereby the severity of labelling and the number of VIP-positive cells were not altered under the influence of various types of asphyxia.

The lack of VIP-positive nerves in the studies from Smith et al. [61] may have arisen from using paraffin-wax embedded tissue, which is not a recommended method of detecting nerve fibers [60]. This thesis is supported by later studies, in which the presence of VIP was noted (similar to other mammalian species) in the varicose nerve fibers of the human carotid body [63]. Nerves in which VIP is often co-localized with neuropeptide Y have been described as one of three types of nerve fibers (besides nerves containing substance P and calcitonin gene-related peptide and processes containing tyrosine hydroxylase and neurofilament 160 KD) supplying the glomus cell clusters. There was a small population of VIP-positive nerves that were characterized by numerous varicosities and encircled the nests of glomus cells but did not penetrate them [63].

### 3.2. Cat

The cat was the first mammalian species in which VIP was found in the nerve fibers located in the carotid body in 1979 [24]. That study described the presence of VIP in the nerve fibers of the feline carotid body and was characterized by numerous varicosities and a patchy distribution between carotid body cells [24]. The highest density of such nerves has been found around blood vessels, and only single VIP-positive processes penetrated the glomus cell clusters. On the other hand, VIP has not been found in cells located within the carotid body [24]. Studies of VIP in the feline carotid body were continued by Wharton et al. [25]. These authors did confirm the earlier observations and found VIP in a dense network of varicose nerve fibers, which were mainly located around blood vessels and type I carotid body cells containing enkephalin. VIP was not found in carotid body cells, but its presence has been confirmed in neuronal cells located in the periphery of the carotid body as well as in nerves supplying carotid arteries [25]. It has also been shown that the amount of VIP in the feline carotid body is relatively high and amounts to above 70 pm/g of carotid body extract [25]. Moreover, it is known that VIP may colocalize with nitric oxide in the same nerves in the cat carotid body and that these nerves may be the processes of both neurons (most likely of parasympathetic origin) located in the ganglia in the immediate vicinity of the carotid body and the sympathetic neurons of the superior cervical ganglion [64].

### 3.3. Rat

**Figure 1:**
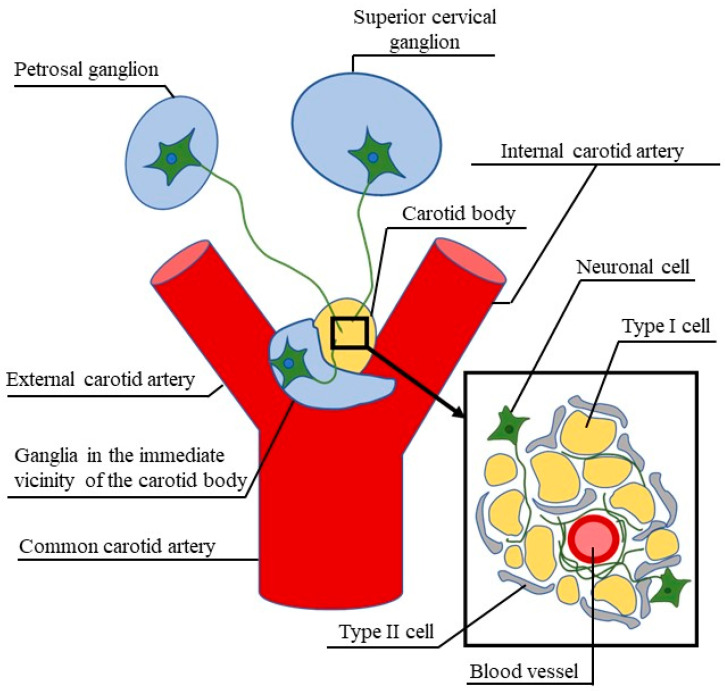
Vasoactive intestinal polypeptide (VIP)-positive neuronal structures (green) supplying the rat carotid body in light of current knowledge.

The rat is a species, in which the presence of VIP in the carotid body has been described in relatively numerous publications. These publications have reported a high number of VIP-positive nerves in the rat carotid body, especially around blood vessels and near the wall of the carotid arteria. They were also found in the parenchyma of the carotid body between the glomus cell clusters [65,66,67]. The majority of these fibers were thin and were characterized by numerous varicosities [65,66,67]. Moreover, neuronal ganglion cells closely associated with the rat carotid body have been described. Some of these cells contained VIP, especially, the ones localized in the entry to the carotid sinus nerve leading to the carotid body and near the nerves coming from the superior cervical ganglion [19]. VIP-positive neuronal cells in the carotid body, together with the fact that the number of nerve fibers containing this peptide did not undergo changes after denervation of the carotid body, suggests the mainly intrinsic origin of VIP-positive nerves in this organ [67]. The observations of Kummer at al. [67] show that the denervation of the rat carotid body did not affect the number of VIP-positive nerves in this organ. This was also confirmed by Dahlqvist et al. [68], who did not observe changes in the number of such fibers after vagotomy or sympathectomy. On the basis of these studies, Dahlqvist et al. [68] reached a conclusion that VIP-like immunoreactive (VIP—LI) nerves in the rat carotid body have a sensory vagal or sympathetic postganglionic origin. In turn, other studies have demonstrated that there are only a few VIP-positive neurons supplying the rat carotid body located in the petrosal ganglia, in which VIP was present in less than 1% of all neurons supplying the carotid body [15,18]. In turn, the presence of VIP in neurons supplying the carotid body and located in the jugular and nodose ganglions has not been noted [18]. The distribution and origin of VIP-positive nerves in the rat carotid body are presented in Figure 1.

Later studies have also described changes in the density of VIP-positive nerves in the rat carotid under chronic three-month hypoxia [65,69]. Kusakabe et al. [66,69] calculated the density of VIP-LI nerves based on the number of VIP-positive varicosities per unit area (10^4^ μm^2^) and calculated that this value under physiological conditions amounted to 12.5 ± 1.8. The number of VIP-LI varicosities was much lower than the number of varicosities containing neuropeptide Y, and their number was similar to the number of calcitonin gene-related peptide-positive nerves and much higher than nerves immunoreactive to substance P [66].

The character and severity of changes in the number of VIP-positive nerves depend on the type of hypoxia. Three types of hypoxia with different levels of carbon dioxide (isocapnic, hypocapnic and hipercapnic) have been described [66,70]. It has been shown that, during chronic isocapnic hypoxia, the number of VIP-positive varicosities per unit area was 1.8 times higher than in control animals [66]. Slightly smaller changes have been noted in chronic hypocapnic hypoxia, during which the number of VIP-LI varicosities was 1.4 times higher than in control animals [66,71]. The higher number of VIP-positive nerves was associated with enlarged vasculature, and hypoxia-induced changes remained one or even two (in the case of a long-lasting hypoxia) months after reoxygenation [20,65,72]. In turn, chronic hypocapnic hypoxia did not cause changes in the number of VIP-LI varicosities in the rat carotid body [66]. Other studies have shown that, during normobaric hypoxia lasting for 14 days, VIP-like immunoreactivity in the rat carotid body increased by 204% in comparison to physiological conditions [73].

Other pathological factors may also change the number of VIP-LI nerves in the rat carotid body. Namely, it has been described that the density of VIP-like immunoreactive nerve varicosities in this organ was lower in the spontaneously hypertensive rats when compared to normotensive Wistar rats, which suggests that VIP may modify the sensitivity of chemoreceptors in the rat carotid body [74].

### 3.4. Guinea Pig

Contrary to other species, VIP-positive nerves in the guinea pig carotid body are rather sparse [19,75]. They are far less numerous than fibers containing neuropeptide Y. On the other hand, few nerves immunoreactive to VIP have also shown the presence of neuropeptide Y, and this type of nerve has been noted mainly near the ascending pharyngeal artery and carotid sinus [75]. VIP in nerves within the guinea pig carotid body did not co-localize with tyrosine hydroxylase, substance P or calcitonin gene-related peptide [75]. Studies with the use of one type of antibody have shown the absence of VIP-LI nerves in the carotid body after sympathectomy, but the use of other antibodies caused various effects in particular animals. Namely, the lack of VIP-positive nerves was noted in five guinea-pigs subjected to sympathectomy, only a few nerves were present in one animal and the number of fibers immunoreactive to VIP increased in two animals [75].

Moreover, sympathectomy changed the neurochemical characterization of VIP-positive nerves. After this operation, the co-localization of VIP and substance P was found in the same nerves while the same co-localization was not observed in control animals under physiological conditions [75]. The co-localization of VIP and substance P may suggest the participation of VIP in the sensory stimuli conduction in the guinea pig carotid body, but on the other hand, the sensory denervation of the carotid body did not affect the number of VIP-positive nerves in this organ [75]. The most visible changes (significant decrease) in the number of VIP-positive nerve fibers were found after the transection of the carotid sinus nerve, which suggests that such nerves have an extrinsic origin and are most likely the branches of the carotid sinus nerve [75].

Other studies have shown the presence of autonomic nerves immunoreactive to VIP in the guinea pig carotid body. These nerves are processes of cells located in the superior cervical ganglion and contain only VIP or VIP with neuropeptide Y [67]. A moderately dense network of VIP-positive nerves were also found in the wall of the carotid arteries [76].

### 3.5. Chipmunk

Information concerning VIP in the innervation of the chipmunk carotid body is limited to one publication [77]. In this species, VIP-positive nerves with numerous varicosities were first observed around blood vessels. Moreover, it was reported that the number of VIP-positive varicosities increased in hibernating animals, which is most likely connected with hypoxia appearing during hibernation, where the respiration and heart rates are decreased [77].

## 4. VIP in the Carotid Body of Birds

The carotid body in birds is located between the nodose ganglion of the vagal nerve and the recurrent laryngeal nerve at the beginning of the common carotid artery [78]. Moreover, glomus cells typical for the carotid body in birds have been found not only in the carotid body but also in the wall of the common carotid artery and within the cranial and caudal parathyroid glands [78,79,80]. It is known that the chicken carotid body is richly supplied with nerve fibers, which are characterized by a high degree of neurochemical differentiation [78,81,82]. Apart from substance P, calcitonin gene-related peptide, somatostatin and galanin, VIP has also been found in the nerves within the carotid body of birds [78,81,82]. In a similar way to mammals, VIP-positive nerves in the chicken carotid body are characterized by numerous varicosities [81]. Firstly, such fibers are located in the connective tissues surrounding the carotid body parenchyma, especially in the capsular connective tissue in the peripheral parts of the carotid body [81]. Moreover, VIP has also been found in numerous nerves around arteries supplying the chicken carotid body [81,82]. In turn, nerves containing VIP in the carotid body parenchyma show a moderate density [81]. VIP has also been described in nerves supplying the glomus cells distributed in the wall of the common carotid artery, but such nerves are not numerous [79]. In turn, the glomus cells located in the cranial and caudal parathyroid glands are supplied by a dense network of varicose fibers containing VIP, which strongly suggests important roles for VIP in the regulation of local blood flow and parathormone secretion [80,83]. In light of previous studies, VIP in the chicken carotid body is involved in chemoreception and circulation [81].

It is also known that the number of VIP-positive nerves in the chicken carotid body is subject to change under various factors. Studies concerning changes in the carotid body innervation have shown that the incision of the 14th cervical ganglion of the sympathetic trunk caused a severe reduction in the number of VIP-positive nerves in the carotid body [82]. In turn, other types of denervation of the chicken carotid body (including nodose ganglionectomy, midcervical vagotomy, cutting the recurrent laryngeal nerve and ganglionectomy of the superior cervical ganglion) did not affect the number of VIP-positive nerves in this organ [82]. These observations have shown that the majority of VIP-positive nerves in the carotid body are derived from the 14th cervical sympathetic ganglion [78,82].

The next factor, which may affect the number of VIP-positive nerves in the chicken carotid body is hypoxia. It was shown that thirty-five days of isocapnic hypoxia caused clear changes in the morphology of the chicken carotid body. Enlargement of the carotid body and atrophy of the connective tissue around the carotid body parenchyma were accompanied by a decrease in the number of nerves immunoreactive to VIP [84].

## 5. VIP in the Carotid Body of Amphibians

In amphibians, chemoreceptors are located in the carotid labyrinth, which is the equivalent of the mammalian carotid body [85]. The presence of VIP (alongside numerous other neuronal active substances including substance P, neuropeptide Y, calcitonin gene-related peptide and somatostatin) has been found in relatively numerous nerve fibers located in the carotid labyrinths of various amphibians species, including *Bufo japonicus, Rana catesbeiana, Rana nigromaculata, Xenopus laevis, Cynops pyrrhogaster* and *Arnbystoma tigrinum* [86,87]. Generally, VIP-positive nerves were thin and characterized (in a similar way to the mammalian and avian carotid body) by numerous varicosities [86]. In all amphibian species, the studied nerves immunoreactive to VIP were found both in the peripheral and central portions of the carotid labyrinth. Such nerves were mainly present in the intervascular stroma, and they are associated with the sinusoidal plexus [86]. On the other hand, clear interspecies differences in the density of VIP-positive nerves were found. The densest network of such fibers were noted in *Rana nigromaculata*, where the fibers located in the peripheral part of the carotid labyrinth were more numerous. In turn, only a few positive nerves immunoreactive to VIP were described in both peripheral and central parts of the carotid labyrinth in *Xenopus laevis* and *Arnbystoma tigrinum* [86].

It is also known that ontogenesis affects the number of VIP-positive nerve fibers in the amphibian carotid labyrinth. Studies on bullfrog (*Rana catesbeiana*) have indicated that VIP-positive nerves in the carotid labyrinth appear at the early metamorphic stage [88], but they are sparse until the completion of metamorphosis. The number of VIP-positive nerves increases from 1 to 5 weeks after metamorphosis, and 8 weeks after metamorphosis, the number and distribution of such nerves are similar to that observed in the adult bullfrog [88]. Moreover, contrary to adult amphibians, the presence of VIP in glomus cells during metamorphosis has also been noted. Results obtained by Kusakabe [88] suggest that VIP is less important in larval development, and its regulatory functions, mainly in vascular regulation, are more relevant after metamorphosis in the carotid labyrinth of the adult animal.

## 6. Functions of VIP in the Carotid Body

The knowledge of the functions of VIP in the carotid body is relatively limited, fragmentary and not clear. As mentioned above, significant interspecies differences in the distribution of VIP in the carotid body strongly suggest that the exact roles of VIP in this organ are different in various animal species. There are many aspects connected with the functioning of VIP in the carotid body.

Previous studies have indicated that VIP affects carotid body chemoreceptors in the cat. Experimentally administered VIP caused changes in spontaneous chemoreceptor discharge, and the character of the influence clearly depended on the dose of the peptide [26]. The administration of small doses of VIP resulted in a decrease of the spontaneous discharge of chemoreceptors located in the carotid body, whereas high doses of this neuropeptide caused the opposite effects (an increase of spontaneous discharge) [26]. On the other hand, it is known that VIP (as opposed to pituitary adenylate cyclase-activating polypeptide—PACAP (see paragraph 7) did not cause stimulation of the type-I glomus cells through an increase of intracellular Ca^2+^ ion concentration, or, if so, the effects of VIP-induced stimulation were very faint [89].

Due to the fact that active substances located in the same nervous structures most often play similar roles, some functions of VIP may be elucidated by analyzing the roles of factors with which it co-localizes within nerves supplying the carotid body. Therefore, the co-localization of VIP and nitric oxide synthase may suggest that the roles of VIP are similar to those performed by nitric oxide, which is known to be a factor that inhibits the activity of carotid body cells [90,91]. It is also known that the inhibition of type-I glomus cells may be realized in various ways, namely by the influence of their excitability as well as by the increase in blood flow and oxygen delivery [92]. In turn, the co-localization of VIP with neuropeptide Y may suggest that VIP (in a similar way to neuropeptide Y) participates in some mechanisms connected with the excitation of the carotid body chemoreceptors [93]. These seemingly contradictory functions of VIP (inhibition and excitation of type-I glomus cells) are in agreement with the abovementioned studies by McQueen and Ribeiro [26], in which both VIP-induced excitation and inhibition of carotid body chemoreceptors were noted and the character of the impact of VIP depended on the dose of this peptide.

In turn, the localization of VIP-positive nerves, the great majority of which have been found near the blood vessels in the carotid body [25,65,66,67], may suggest that VIP regulates the carotid body activity by influencing the blood flow. This is very likely, since VIP is known as a strong vasodilatory factor in various internal organs [41]. Moreover, the increase in the number of VIP-positive nerves in the carotid body under hypoxia together with the hypoxia-induced enlargement of the carotid body vasculature may indicate that the main way VIP-dependent regulatory processes of the carotid body functions are connected with the regulation of the vascularization, leading to an increase in the blood flow and oxygen delivery but not by directly impacting the type-I glomus cells [94].

## 7. Pituitary Adenylate Cyclase-Activating Polypeptide in the Carotid Body

While considering VIP in the carotid body, pituitary adenylate cyclase-activating polypeptide (PACAP) should also be mentioned. It is the second member of a glucagon/secretin superfamily which has been found in this organ. PACAP, which is characterized as having 68% sequence homology with VIP, has been noted in the type-I glomus cells, where specific G-protein coupled receptors for this peptide—PAC1—have also been found [95]. It should also be pointed out that PACAP, apart from PAC1, may interact with VPAC1 and VPAC2, whereby VPAC1 and VPAC2 bind VIP and PACAP with equal affinities. Moreover, PAC1 binds PACAP with 100–1000 times higher affinity than VIP [30,41,89].

PACAP is a factor for which the influence on the activity of type-I glomus cells is relatively well known. It has been shown that intravenous administration of PACAP results in the clear stimulation of breathing and an increase of ventilation [96]. On the other hand, these PACAP effects have not been noted after the bilateral cutting of the carotid sinus nerve, which strongly suggests that this peptide stimulates respiration through the influence of peripheral chemoreceptors located in the carotid body [96]. Other studies of this issue have shown that PACAP-deficient animals are characterized as having defects in respiratory control, manifesting itself in a clear reduction in ventilation and an impaired response to hypoxia and hypercapnia, which in turn leads to apnea and sudden neonatal death [97]. These studies have strongly suggested key roles for PACAP in the regulation of carotid body functions.

Subsequent studies have confirmed these observations and have shown that PACAP stimulates type-I glomus cells through the impact on PAC 1 receptors [89,98,99]. Initially, it was thought that the entire pathway by which PACAP affects type-I glomus cells is an adenylate cyclase-protein kinase A (PKA) pathway [89,98]. The stimulation of this pathway leads to a reduction in TASK-1-like K^+^ currents, an increase in intracellular Ca^2+^ ion concentration and glomus cell depolarization [89]. Moreover, some mechanisms observed during the impact of PACAP on the carotid body (namely the increase of intracellular Ca^2+^ concentration dependent on the presence of extracellular Ca^2+^ and a reduction of tetraethylammonium (TEA)-insensitive current) were similar to those observed during hypoxia [98], which suggests that PACAP is involved in the processes occurring during hypoxia. However, the most recent studies have proven that PACAP may affect the carotid body chemoreceptors by various signaling pathways, mainly including phospholipase C (PLC) and protein kinase C (PKC)—pathways leading to the activation of transient receptor potential cation (TRPC) and/or T-type channels and the inactivation of voltage-gated potassium (Kv) channels [99].

The important functions of PACAP in the regulation of type-I glomus cell activity during hypoxia can be testified by the fact that the number of carotid body cells containing both PACAP and PAC1 is significantly increased during chronic and intermittent hypoxia [95]. Moreover, both types of hypoxia have been shown to cause an increase in the mRNA level of PAC1 in type-I glomus cells [95].

## 8. Conclusions

In light of previous studies, VIP seems to be an important factor involved in regulatory processes of the carotid body. The presence of this peptide has been described in numerous species, including not only mammals but also birds and amphibians [19,78,87]. The current knowledge of the distribution of VIP in the carotid body is summarized in Table 1.

Firstly, VIP was described in the nerve fibers of the carotid body, which are especially located in the connective tissue between the carotid glomus cell clusters and near blood vessels. Besides interspecies differences, VIP has been described mainly in thin nerves with numerous varicosities, although some investigations have described VIP in the carotid body parenchyma cells and neurons in the ganglia adjacent to the carotid body [19,61,75,78]. VIP-positive nerves in the carotid body are processes of neuronal cells located in the petrosal ganglion and superior cervical ganglion as well as neurons located in the immediate vicinity or inside of the carotid body (Figure 1). It is known that VIP in the carotid body may be involved in the regulation of parenchymal cell activity, but the main functions of VIP in this organ seem to influence blood flow [26,67,74]. It may also participate in adaptive processes within the carotid body, and the number and distribution of VIP-positive nerve fibers may undergo changes in response to physiological and pathological stimuli, including various types of hypoxia or hypertension [72,75,88]. Nevertheless, many aspects connected with the distribution and functions of VIP in the carotid body still remain unclear. First of all, the exact mechanisms of the influence of VIP on the carotid body as well as the distribution of VIP receptors in this organ should be explained in further studies. Moreover, VIP is considered a promising therapeutic factor in some diseases. Therefore, the key questions that need to be explored are connected with the possibility of using VIP as a regulatory factor in the functioning of the carotid body to maintain cardiorespiratory homeostasis and to potentially remedy hypertension, systolic heart failure and other disorders in which chemoreceptor activity plays an important role. Unfortunately, the majority of studies concerning this issue are from the 1980s and 1990s and the problems connected with VIP activity in the carotid body seem to be marginalized in current studies.

## Figures and Tables

**Table 1:** The presence of VIP in the carotid body summary of current knowledge.

Species	Structure	Reference
Human	Glomus cells Nerve fibers	[61,62] [63]
Cat	Nerve fibers Neuronal cells	[24,25] [25]
Rat	Nerve fibers Neuronal cells	[19,65,66,67,68,69,74] [67]
Guine pig	Nerve fibers	[19,67,75]
Chipmunk	Nerve fibers	[77]
Mouse	Nerve fibers	[19]
Monkey	Nerve fibers	[58]
Chicken	Nerve fibers	[78,81,82,84]
Amphibians: *Bufo japonicas* *Rana catesbeiana* *Rana nigromaculata* *Xenopus laevis* *Cynops pyrrhogaster* *Arnbystoma tigrinum*	Nerve fibers	[86,87,88]
